# In Memoriam —John S. Clark, D. D. S

**Published:** 1867-04

**Authors:** 


### IN MEMORIAM.—JOHN S. CLARK, D. D. S.

At a meeting of the Dentists of St. Louis, December 13, 1866, called for the purpose of taking suitable action in regard to the death of Dr. John S. Clark, a committee of four having been appointed, presented the following preamble and resolutions, which were unanimously adopted:

Whereas, It has pleased the Supreme Arbiter of the Universe to remove from among us Dr. J. S. Clark, one of the pioneers of Dental Progress in the West, we feel that we have truly lost one of our most talented, energetic and useful members; and being desirous that his labors and sacrifices in behalf of the science should be duly acknowledged, and that his claim to the honor of having been chiefly instrumental in bringing into notice and perfecting valuable improvements in Dental practice should be fully vindicated and handed down to posterity as a part and parcel of the history of the profession.

Dr. Clark was born in Brooklyn, Conn., in 1813, and received his literary education in that State. He moved to St. Louis in 1840, attended lectures at the Medical Department of the State University of Missouri, and turning his attention to Dentistry, soon ranked among the first operators of that time. By invitation of the Faculty of the St. Louis Medical College, he delivered some lectures at the College to the medical students of that institution upon Dentistry. He practiced his profession in St. Louis till 1850, when he removed to New Orleans, where he soon took rank as the first operator in the city. In 1856, he commenced publishing the "*Dental Obturator*," one of the very best Dental publications that has been produced. He took his degree as Doctor of Dental Surgery at the Ohio College of Dental Surgery in 1851, was a member of the American Dental Association, and originated several valuable improvements in Dental practice. He returned to St. Louis in 1865, in poor health, but with undiminished energy, and commenced practice again in this city; but his labors were short, as he was called to his eternal home on the 29th of November, 1866, after a short illness of a week. His energy, perseverance and devotion to the cause of Dental Progress had endeared him to the profession, who feel that the place made vacant by his demise can never be refilled; therefore,

*Resolved*, That we regard the death of our co-laborer, John S. Clark, as a great calamity to the profession in which he had so long occupied a prominent and honorable position.

*Resolved*, That to Dr. John S. Clark the profession is, to a great extent, indebted for the successful and philosphical mode of practice now generally adopted for the extirpation of the pulps from the fangs of teeth by the use of broches, and filling the canals with gold, and also for the special elucidation of the process of plugging cavities with gold, performed by being rolled into cylinders and cones.

*Resolved*, That we tender the family of the deceased our heartfelt sympathies; and, though we mourn with them the loss of our professional brother and friend, we recognize in this afflicting dispensation only the accomplishment of the design of the Divine Creator, and bow with uncomplaining submission to the will of Him who doeth all things well.

*Resolved*, That these proceedings be published in the *Missouri Democrat*, and copies, in gilt letters, on mourning paper, furnished the family.

A. D. Sloan,<br>

H. J. McKellops,<br>

H. Judd,<br>

W. N. Morrison, } *Com.*

